# The Argus II prosthesis facilitates reaching and grasping tasks: a case series

**DOI:** 10.1186/1471-2415-14-71

**Published:** 2014-05-23

**Authors:** Aachal Kotecha, Joe Zhong, David Stewart, Lyndon da Cruz

**Affiliations:** 1 NIHR Biomedical Research Centre for Ophthalmology, Moorfields Eye Hospital NHS Foundation Trust and UCL Institute of Ophthalmology, London, UK; 2 Medical Retina and Vitreo-Retinal Service, Moorfields Eye Hospital NHS Foundation Trust, London, UK

**Keywords:** Retinal prosthesis, Functional vision, Reach and grasp

## Abstract

**Background:**

To evaluate the reach-to-grasp performance of patients fitted with an epiretinal artifical retina device.

**Methods:**

This was a hospital-based case series consisting of six patients fitted with the Argus II (Second Sight Medical Products Inc, California, USA) retinal prosthesis. Participants were asked to reach out and pick up a high-contrast cuboid object with the prosthesis in the ‘On’, ‘Off’ or ‘Scrambled’ setting presented in a randomised order. The ‘Scrambled’ setting consisted of a random, scattered signal presented to the prosthesis. The session was repeated after a 4–6 week period. Hand movements were measured using motion detection cameras. The number of successful object grasps was calculated.

**Results:**

The number of successful grasps was greater with the prosthesis in the ‘On’ setting (visit 1: median [interquartile range] percentage success: ‘Off’ = 0 [0 to 50]%, ‘On’ = 69 [67 to 95]%, ‘Scrambled’ = 59 [42 to 95]%; Friedman Chi-squared test statistic 6.5, p = 0.04; visit 2 median [IQR] percentage success: ‘Off’ = 0 [0 to 25]%, ‘On’ = 69 [53 to 100]%, ‘Scrambled’ = 28 [13 to 63]%; Friedman Chi-squared test statistic 8.4, p = 0.02).

**Conclusions:**

The use of an electronic retinal prosthesis facilitates reach-and-grasp performance. Further work should explore how performance can be improved with targeted rehabilitation.

## Background

Retinitis pigmentosa (RP) is the most common of the inherited retinal diseases with a prevalence of approximately 1 in 4000 [1]. The condition results in a progressive degeneration of the photoreceptive layer of the retina that can lead to a complete loss of vision [2], and is responsible for ~3% of all blindness registrations in the UK [3]. Post-mortem studies suggest that although the disease damages the photoreceptor layer, there is moderate preservation of the inner nuclear layer, and relative sparing of the ganglion cell layer [4,5], with evidence of significant changes in interconnectivity between cells of different layers [6]. Studies on human subjects with RP have shown that electrical stimulation of localised retinal areas elicits visual percepts (‘phosphenes’) in spatially correlated areas [7-9]. These findings have led to the development of long-term implantation of retinal prostheses that aim to stimulate the residual inner retina [10].

Retinal prostheses have been shown to facilitate an improvement in letter identification [10,11], and improve the performance of both spatiomotor ‘pointing’ tasks [12] and motion detection [13]. As such, they have become a potential treatment option for patients with RP.

The Argus II retinal prosthesis, developed by Second Sight (Second Sight Medical Products Inc, California, USA), is the first retinal prosthesis to receive regulatory approval, obtaining a European CE mark in June 2011 and US Food and Drug Administration approval in February 2013. Visual information is captured by a spectacle-mounted video camera, and a video processing unit converts the information into a digital signal that stimulates the implanted electrode array.

Patients implanted with the device are trained to develop ‘camera-to-hand’ co-ordination, such that they are able to detect an object and accurately point to its position in space [12]. However, the ability to perform reaching to grasp tasks has not yet been reported. The act of reaching to grasp an object is a basic task performed many times a day and is facilitated by vision. Information about the object’s spatial location is necessary for fast and accurate reaching, whilst an assessment of the object’s intrinsic properties, such as its size, shape and weight, is required to both pre-shape the grasp posture and select the most appropriate contact points to ensure a stable grip [14,15]. Reach and grasp movements are a useful indicator of functional performance in patients with visual impairment and have been previously evaluated in patients with ocular disease [16-18].

The purpose of this study was to evaluate the reach-to-grasp movements of a subset of patients implanted with the Argus II electronic prosthesis.

## Methods

The Argus II epiretinal prosthesis consists of a 60 electrode microarray covering a 20 degree area of the central visual field. An external video camera mounted on a pair of spectacles wirelessly delivers information to the microelectrode array via an inductive radio frequency coil link embedded in the superotemporal sclera. Safety data for all patients participating in the multi-centre international trial of the device have been reported [19,20], and suggest that the device has a good safety profile. For this exploratory study, patients recruited from the Moorfields Eye Hospital NHS Foundation Trust site in London, UK, participated. The Argus™ II Retinal Simulation System Feasibility study had local research ethics committee approval (Moorfields and Whittington local research ethics committee). Informed consent, according to the Tenets of the Declaration of Helsinki, was given to all patients prior to participation in the trial.

### Patient selection

Seven patients have been implanted with the retinal prosthesis at the Moorfields centre (all in the right eye) and their demographic details are detailed in Table 1. One patient (id 51002) declined to participate in the present study, thus 6 patients were involved in the test procedure. All participants had the prosthesis implanted for a minimum of 3 years prior to participating in the present study.

**Table 1:** Demographics of patients implanted with Argus II prosthesis at the Moorfields Eye Hospital NHS Foundation Trust centre

**ID**	**Diagnosis**	**Date of implantation**	**Gender**	**Age at implantation (years)**	**VA at time of implantation (logMAR)**
51001	RP aged early 20s	April 2008	M	70.7	R:B LP
					L: BLP
51002	RP aged 16 years	April 2008	M	51.3	R: BLP
					L: BLP
51003	RP aged 19 years	June 2008	M	72.3	R: BLP
					L: BLP
51005	RP aged 7 years	March 2009	M	55.8	R: <2.9
					L: <2.9
51006	Choroideraemia aged 46 years	April 2009	M	66.7	R: <2.9
					L: <2.9
51007	RP aged 28 years	June 2009	M	63.1	R: <2.9
					L: <2.9
51009	RP aged 11 years	August 2009	F	45.3	R: <2.9
					L: <2.9

Key: ID: identification number, VA = visual acuity, RP = retinitis pigmentosa, BLP = bare light perception.

### Experimental conditions

**Figure 1:**
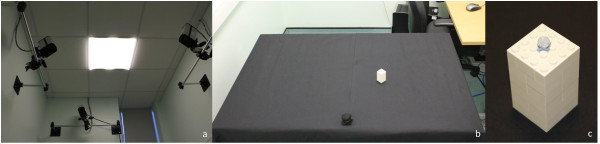
**The experimental set-up. (a)** Three ProReflex motion capture units triangulated the workspace at a height of approximately 1.5 metres above the table; **(b)** the experimental set up consisted of a table covered in black felt, illuminated uniformly from above; **(c)** the participant was instructed to reach and grasp a white Lego® block.

The experimental setup consisted of a table covered with matt black felt illuminated uniformly from above. Three motion-capture units (ProReflex, Qualisys AB, Sweden) triangulated the area at a height of approximately 1.5 metres above the table. Three lightweight infrared (IR) reflective markers of approximately 7 mm diameter were placed on the participant’s preferred hand, determined using the Edinburgh handedness questionnaire [21], for recording its movement in three-dimensional space [See Figure 1]. One marker was attached to the wrist using a Velcro strap and two were placed on the participant’s opposing distal borders of the thumbnail and index fingernail using Blu-Tack®. Movements of the IR markers were tracked by the motion-capture units and recorded directly by a personal computer based system.

Participants were required to reach out and grasp a high contrast cuboid object, dimensions 30 mm by 30 mm by 60 mm made of white Lego®, positioned near (250 mm) or far (450 mm) at 20 degrees from the start position in either the ipsilateral or contralateral hemispace relative to the reaching hand ( 4 positions in total). These positions were chosen to allow for as natural a movement as possible, such that the participant did not have to overstretch their arm in order to reach the object, and because we did not wish for the object to be in the video camera’s ‘line of sight’ at the start of the movement.

### Exploration of reach-to-grasp ability

Prior to the start of the experimental procedure, participants were given standardised instructions for the task requirement. With the prosthesis switched off, participants were first asked to feel across the table with their hands to establish the dimensions and edges of the workspace, after which they were required to handle the object to familiarise themselves with the size, shape and weight. The participants were advised of the colour of the table and object. The retinal prosthesis was then switched on and the object was placed in the line of ‘sight’ of the video camera for the participant to establish the appearance of the object as imaged by the device. The object was then placed at the edges of the table for the participant to establish the maximum limits of head/camera movement required for the test procedure. Participants were advised that during the test procedure, the prosthesis would be set to one of 3 settings: ‘On’, where the prosthesis was set to standard function, ‘Off’, where it was switched off completely and ‘Scrambled’ where the microelectrode array was fully functioning but the signal input would result in the electrodes being stimulated in a random, scattered pattern. The prosthesis settings would be presented in a randomised order to which the participant would be masked. The participant was advised that they would undertake the test procedure under one of 2 eye conditions, eyes ‘patched’ where both eyes would be taped closed or ‘unpatched’, and that they would be given a time frame of 30 seconds in which to find the object, reach out and bring it to the start position. Participants were advised that the object would be placed in one of 4 positions on the table. The purpose of ‘patching’ the eye was to eliminate the use of any residual vision. The 30 second recording window was chosen arbitrarily to try and maximise the participants’ chances of completing the task, without being so excessive as to result in fatigue over the course of the experimental procedure.

Participants were instructed to locate the object, pick it up and bring it to the start position. Prosthesis setting, eye condition and object position were presented in a randomised order (list generated by http://www.randomizer.org prior to the start of the test procedure), with participants masked to object placement at the start of each recording. Participants were given an audible instruction as to when to start locating the object (‘start now’) and were advised when the 30 second recorded period terminated. Participants performed 48 ‘reach-and-grasp’ movements in total, (3 prosthesis settings: On, Off, Scrambled; 2 eye settings: Patched, Unpatched; 8 repetitions: 4 in the ‘near’ and 4 in the ‘far’ locations) in total. All tests were performed by a single observer (AK).

Each patient was retested after a period of 4–6 weeks to examine the repeatability of movements. The study was performed between October 2011 and October 2012, determined by the availability of the patients.

### Movements quantified

Each IR –marker generated x,y,z co-ordinate outputs for each 30 second recording and were analysed using purpose written software (Matlab 7.4.0.287 R2007a, The Mathworks Inc, Massachusetts, U.S.A). Definitions of reach parameters calculated are given in Table 2. Reach accuracy was evaluated using the ‘path deviation ratio’ (PDR), with a ratio of 1 indicating a direct hand trajectory to the object on the table, and the ‘time to object contact’ (TTC), with a shorter time indicating a rapid reach to the object.

**Table 2:** Definitions of data quantified

**Kinematic parameter**	**Definition**
*General kinematics*	
Movement onset (MO; seconds)	Time at which wrist speed exceeded 50 mm/s
Object contact (seconds)	When object movement ≥ 1 mm from original position
*Reach dynamics*	
Time to object contact (TTC; seconds)	Time between movement onset and object displacement ≥ 1 mm
Path deviation ratio	Deviation of the movement trajectory from a straight route between the starting and object contact wrist positions

### Data analysis

The percentage of completed movements, defined as a successful reach and grasp of object within the 30 second trial period against the number of trials undertaken, was calculated for each participant.

The data followed a non-normal distribution and thus the non-parametric Friedmann and Wilcoxon tests were used to evaluate differences in reach and grasp movements.

## Results

### Successful grasps

**Figure 2:**
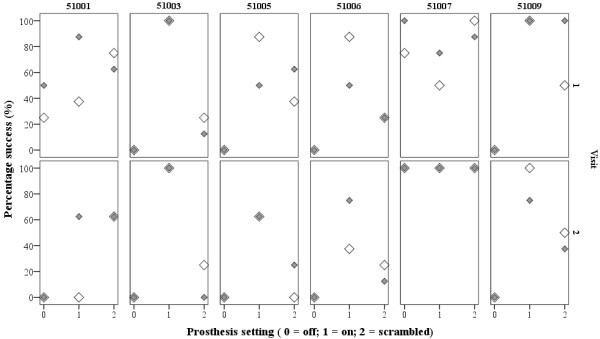
**The percentage of successful grasps obtained for each patient within each trial condition.***Shaded symbols = eyes patched, clear symbols = eyes unpatched.* The data show that, for the majority of participants, more successful grasps were achieved when the prosthesis was activated.

Figure 2 shows the percentage of successful grasps for each patient at the 2 visits.

At the first visit, there were significant differences in success rate under the 3 different settings (patched and unpatched data pooled median [interquartile range] percentage success: ‘Off’ = 0 [0 to 50]%, ‘On’ = 69 [67 to 95]%, ‘Scrambled’ = 59 [42 to 95]%; Friedman Chi-squared test statistic 6.5, p = 0.04). This was maintained at the second visit (‘Off’ = 0 [0 to 25]%, ‘On’ = 69 [53 to 100]%, ‘Scrambled’ = 28 [13 to 63]%; Friedman Chi-squared test statistic 8.4, p = 0.02). Wilcoxon test analyses showed that success rates between ‘Off’ and ‘On’ were significant (‘Off’ v ‘On’ visit 1: Z statistic = −2.0, p = 0.046; visit 2: Z statistic = −2.0, p = 0.042; ‘Scrambled’ v ‘On’ visit 1: Z statistic = −0.7, p = 0.5; visit 2: Z statistic = −1.8, p = 0.08).

### Quantification of kinematic parameters

**Figure 3:**
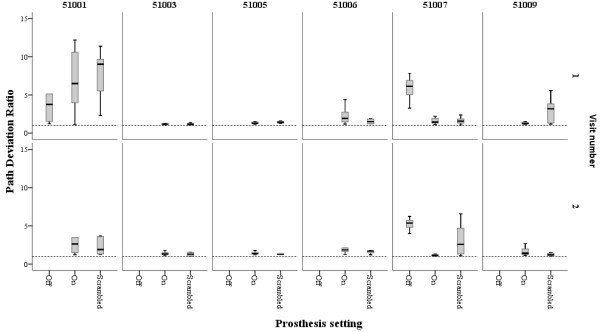
**Path deviation ratio for each successful grasp obtained by each patient.** The path deviation ratio is the ratio between the path taken by the hand to reach the object and the shortest straight line distance between the hand and object at the start. The path trajectory was measured using the wrist marker. The dotted line represents a ratio of 1.0, which would indicate a straight line trajectory of the reaching hand to the object. *Boxes represent median, 25th and 75th percentiles, error bars 5th and 95th percentiles of successful grasps under each prosthesis setting; patched and unpatched data pooled.*

**Figure 4:**
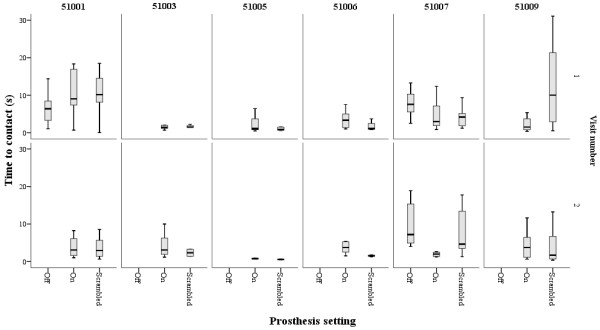
**Time to object contact (TTC) for each successful grasp obtained by each patient.** TTC represents the time between the onset of hand movement to making contact with the object, defined as the object moving ≥1 mm from its position on the table. The shorter the TTC, the quicker the hand reached the object. *Boxes represent median, 25th and 75th percentiles, error bars 5th and 95th percentiles of successful grasps under each prosthesis setting; patched and unpatched data pooled.*

Kinematic parameters were calculated for successful grasps; Figures 3 and 4 show the TTC and PDR for each subject at the 2 visits. At visit 1, only 2 participants made successful grasps with the prosthesis in the ‘off position’ (51001 and 51007); at visit 2 only 1 participant succeeded in grasping the object with the prosthesis off (51007). Thus, for the rest of the analysis, the differences between prosthesis setting ‘on’ and ‘scrambled’ were studied. There were no significant differences between ‘patched’ and ‘unpatched’ eye conditions within each prosthesis setting, therefore data were pooled. The median for each patient under the ‘on’ and ‘scrambled’ settings were calculated. Wilcoxon ranks tests showed no significant differences in MO at either visit, although there was a non-significant trend for movement onset to occur sooner in the ‘On’ setting compared with ‘Scrambled’ at visit 2 (visit 1 median [IQR] MO: ‘On’ = 10.6 [8.1 to 13.8] seconds, ‘Scrambled’ = 12.4 [10.4 to 18.5] seconds, z-statistic = −1.4, p = 0.2; visit 2 median [IQR] MO: ‘On’ = 10.6 [7.6 to 15.2] seconds, ‘Scrambled’ = 16.3 [8.1 to 21.5] seconds, z-statistic = −1.8, p = 0.08). There were no significant differences in TTC and PDR between ‘On’ and ‘Scrambled’ settings at either visit (visit 1 median [IQR] TTC: ‘On’ = 2.3 [1.4 to 4.8] seconds, ‘Scrambled’ = 3.0 [1.1 to 10.1] seconds, z-statistic = −0.9, p = 0.3; PDR: ‘On’ = 1.4 [1.2 to 3.1], ‘Scrambled’ = 1.5 [1.4 to 4.6], z-statistic = −1.1, p = 0.2. Visit 2 median [IQR] TTC: ‘On’ = 3.1 [1.7 to 3.8] seconds, ‘Scrambled’ = 2.0 [1.3 to 3.4] seconds, z-statistic = −0.9, p = 0.3; PDR: ‘On’ = 1.4 [1.2 to 3.1], ‘Scrambled’ = 1.5 [1.3 to 4.6], z-statistic = −0.9, p = 0.3).

## Discussion

The results of this study show that the ability of individuals with profound loss of vision to reach and grasp a table top object is facilitated with an artificial retinal prosthesis. With the prosthesis in the ‘On’ position, there were more successful grasps and a greater accuracy of reaching movements compared with when the prosthesis was in the ‘Off’ setting, suggesting that use of the prosthesis may facilitate reaching and grasping movements.

Two participants (51001 and 51007) made successful reaches with the prosthesis in the ‘Off’ setting. Participant 51007 exhibited larger path deviation ratios and longer times to object contact under this setting, suggesting a less efficient movement overall. This may be explained by the ‘search’ tactic used by this participant when reaching for the object with the prosthesis ‘Off’; the participant used his hand to ‘scan’ across the table until the object was found. In contrast, his reaches made with the prosthesis in the ‘On’ setting had shorter time to contact and path deviation ratios, indicating that he made a more efficient movement towards the object in this setting. However, participant 51001 displayed very variable movements in all prosthesis settings. This participant is known to have specific difficulties when using the prosthesis, in that he suffers a loss of connection between the implanted radio frequency coil and head mounted camera, thought to be related to an acquired, intermittent onset, exotropia.

Under the ‘On’ and ‘Scrambled’ settings movement onset time occurred after a delay of over 10 seconds after recording started, and is likely to reflect an attempt by the participant to detect the object position using the prosthesis prior to attempting the reach. This represents a considerable delay when compared with reaching behaviour studies of patients with acquired loss of central [18,22] and peripheral [17] vision.

For 2 participants (51007 and 51009) the path deviation ratio was slightly longer and more variable under ‘Scrambled’ setting, suggesting that in spite of having knowledge that an object was present on the table, there was insufficient information to make a precise movement towards it. However, this was not the case with the remaining participants, and suggests that for this study, use of the prosthesis was primarily to detect the location of the object to facilitate the reach. Under the ‘Scrambled’ setting, when the video camera detects an object on the table, it sends a random signal to the microarray, and therefore will not give full information of the ‘form’ of the object, just an indication that an object is present. The primary aim of the study was to investigate whether use of the prosthesis facilitated reaching to a table-top object; greater differences between the ‘On’ and ‘Scrambled’ settings may occur if participants are required to differentiate between forms of objects in their reach. Previous work evaluating letter recognition tasks clearly show that form recognition is superior with the prosthesis in the ‘On’ setting over the ‘Scrambled’ setting [11].

Once the movement was initiated, time to object contact was achieved within 1.5 to 3 seconds. Castiello [23] studied the reach and grasp movements of congenitally blind patients and found that the time to object contact was approximately 1 second. However, a key difference between his and the present study is that his subjects were informed of the location of the object prior to recording movements. Castiello wished to evaluate whether there was a decoupling of the reaching and grasping components in blind subjects, whilst the present study aimed to look at reach efficiency using the prosthesis; to achieve this aim no prior knowledge of the object’s position was made available, thus it is difficult to directly compare the findings of these studies. In their study of patients with bilateral central scotomas from age-related macular degeneration, Timberlake et al. [18] found time to object contact durations to be between ~ 0.7 and 1.2 seconds, and similar times to contact have been found in other studies of patients with macular disease [22,24]. The times to object contact in the present study were longer by comparison and may reflect either a lack of confidence in using the prosthesis and the possible difficulties in achieving ‘camera-hand’ co-ordination, or perhaps illustrate the detrimental effect of the lack of visual feedback of hand position to facilitate online control of movement, leading to a more tentative reach movement. In studies of normally sighted individuals where the view of the reaching limb was obscured, other observers have noted longer movement durations [25]. It is thought that without visual information of the hand and arm in space, one is unable to control a ‘reach’ movement precisely [26]. An efficient reach requires information of the hand’s position relative to the object, [25,27] and online control of reaching movements has been found to be deficient in artificially [15,28] and pathologically [17] restricted fields of view. However, in his study of congenitally blind subjects Castiello [23] found that visual feedback was not necessary for transporting the hand to an object, but was important for the scaling of grip aperture. It may be that late-blind participants in this study had already developed strategies to compensate for their lack of visual feedback to function in their every-day life [29] and thus the lack of hand visualisation did not completely prevent them from making an accurate reach. Furthermore, there is some evidence to suggest that it is not so much vision *per se*, but direction of gaze and head position that augments proprioceptive feedback in reaching tasks [30]. Thus, once the object had been located by the prosthesis, the reach was made based on the head and eye position. However, this is speculation and further studies with patients implanted with retinal prostheses should explore this topic further.

Limitations to this study include the laboratory environment and choice of high contrast object; how our findings translate to ‘real-world’ environment remains to be elucidated. Furthermore, the cohort only consisted of the 6 UK participants of the international trial. However, the aim of this study was to evaluate whether reach-to-grasp movements might be used to assess the functional performance of the artificial retina, and the results of this study suggest it may be a useful tool. Future work will evaluate reaching movements to multiple objects to evaluate both object discrimination and the effects of crowding on reach accuracy.

## Conclusions

In conclusion, this study shows that the Argus II retinal prosthesis facilitates reaching and grasping movements in blind persons.

Finally, the most pertinent observation from this pilot study was the delay in actual movement onset. This suggests that whilst use of the prosthesis facilitates reaching for a table top object, movements are not initiated swiftly. It is clear that this should be addressed when considering the rehabilitation strategies for prosthesis usage in this patient group. Future research directions should focus on rehabilitation and use of prosthesis for every-day tasks.

## Competing interests

None of the authors have any competing interests to declare. Mr. daCruz receives funding from Second Sight Medical Products to support the Argus II trial; he has no financial interest in Second Sight Medical Products.

## Authors’ contributions

AK: study conception and design, data acquisition, data analysis, manuscript drafting and final approval; JZ: data acquisition, software development, manuscript final approval; DS: data acquisition, manuscript final approval; LdC study conception and design, manuscript revision, final approval. All authors read and approved the final manuscript.

## Pre-publication history

The pre-publication history for this paper can be accessed here:

http://www.biomedcentral.com/1471-2415/14/71/prepub
